# THE SIGNALING PATHWAY OF SRF-SIRTUIN-2 GENE AND MITOCHONDRIAL FUNCTION

**DOI:** 10.1093/geroni/igae098.2633

**Published:** 2024-12-31

**Authors:** Xiaomin Zhang, Gohar Azhar, Fathima Ameer, Jeanne Y Wei

**Affiliations:** University of Arkansas for Medical Sciences, Little Rock, Arkansas, United States; University of Arkansas for Medical Sciences, Little Rock, Arkansas, United States; University of Arkansas for Medical Sciences, Little Rock, Arkansas, United States; University of Arkansas for Medical Sciences, Little Rock, Arkansas, United States

## Abstract

**Background:**

The sirtuin-2 (SIRT2) is one of the seven sirtuin proteins that deacetylate histones and other non-histone proteins. SIRT2 is localized mainly in the cytoplasm, but it may shuttle into the nucleus. SIRT2 regulates cellular metabolism, growth, stress response and aging. However, the transcription factors that regulate the SIRT2 gene have not been fully elucidated. Here we report the identification of two novel binding sites for the serum response factor (SRF) in the SIRT2 promoter, and the effect of SIRT2 gene expression on mitochondrial function.

**Methods:**

The transcription factor binding sites in the SIRT2 promoter were analyzed with bioinformatic tools, including TRANSFAC, TFBIND and were verified with sequence analysis. SH-SY5Y cells were cultured in either regular serum or low serum medium to simulate an ischemia condition, respectively. The mitochondrial function was measured with a Seahorse XFe96 Analyzer.

**Results:**

and conclusions We have identified two novel SRF binding sites in the human SIRT2 gene promoter, indicating that SRF binds to the SIRT2 promoter, and may recruit SRF cofactors to regulate the SIRT2 gene. SIRT2 plasmid transfection affects mitochondrial function. In the simulated ischemia condition, SIRT2 expression increased the mitochondrial oxygen consumption rate (OCR), while reducing the glycolysis rate (ECAR), suggesting that SIRT2 improves oxidative phosphorylation and protects cells from ischemia. In addition, the biological activators and inhibitors of the Rho/SRF signaling pathway could be used to modulate SIRT2 expression, thereby regulating mitochondrial function in aging and senescence.

